# Farmer social networks: The role of advice ties and organizational leadership in agroforestry adoption

**DOI:** 10.1371/journal.pone.0255987

**Published:** 2021-08-10

**Authors:** Tian Lin, Aung Phyo Ko, Maung Maung Than, Delia C. Catacutan, Robert F. Finlayson, Marney E. Isaac

**Affiliations:** 1 Department of Geography and Planning, University of Toronto, Toronto, Canada; 2 RECOFTC—The Center for People and Forests, Bangkok, Thailand; 3 World Agroforestry (ICRAF), Bogor, Indonesia; 4 Department of Physical and Environmental Sciences, University of Toronto Scarborough, Toronto, Canada; Szechenyi Istvan University: Szechenyi Istvan Egyetem, HUNGARY

## Abstract

With the decline in public budgets for agricultural extension support, ties between members of farmer groups are becoming more important to facilitate information transfer about agroforestry. This paper examines the role of social network ties in predicting organizational leadership in an agroforestry-based farmer group. Using social network data derived from interviews with members of farming groups based in the Ayeyarwady Delta of Myanmar, we established a positive relationship between advice-seeking ties and organizational leadership. In other words, farmers who were highly sought for agroforestry advice were more likely to be elected as leaders of the farmer group. Results show the frequency of interactions through advice-seeking ties also had a positive influence on the probability of farmers holding leadership positions. We found a core–periphery structure for the advice networks, whereby farmer leaders were overrepresented at the network core. Interestingly, general members of the farmer group were also in the core of the core-periphery structure, suggesting that engaging with farmers without leadership roles can also effectively disseminate agroforestry information to peripheral farmers. We conclude that farmer groups are valuable in agroforestry adoption and persistence and further analyses of formal leadership structures are needed to support more transparent and accountable governance.

## Introduction

The shift from top-down technocratic agricultural extension to decentralized, pluralistic farmer-led extension has placed socio-political considerations at the forefront of service delivery [1, 2]. Farmer groups and cooperatives are playing significant roles in alleviating the void of public agricultural extension to provide community-based solutions [1]. Simultaneously, there is a surge of interest and support for the transformation to sustainable agriculture, such as agroforestry systems. These systems draw heavily on knowledge exchange and social relationships to mitigate the risk associated with adoption [3–5].

To overcome resource constraints, such as seeds [6] and knowledge [7, 8], farmers interested in agroforestry have formed farmer groups to serve as vehicles for peer-to-peer information exchange and resource mobilization [9]. Based on the organizational structure of such farmer groups, elected leaders can play pivotal roles in the resource flow on agroforestry [10]. Arguably, elected leaders wield legitimate power and recognized authority in disseminating information [11], thereby potentially determining the uptake or cessation of agroforestry. Through democratic decentralization, electoral processes frame the farmer group as an active site for legitimized negotiation of power, reinforcing informal advice ties as important factors in leadership selection [11–13].

In the midst of decentralized extension reforms, Rivera and Alex have argued the effectiveness of local farmer groups requires strong leadership and highlighted the relational processes for agricultural and rural development [14]. Cook et al. suggest community farmer organizations, including farmer groups and cooperatives, also grant farmers with an avenue for political representation [1], embedding Swanson’s description of “basic building blocks of democratic institutions” [15, p. 16]. Farmers in leadership positions can sanction behaviour as well as defend the collective vision and manage internal disagreements to build the group’s identity [16].

Despite studies showing that knowledge exchange supports the implementation and continuation of agroforestry practices [3, 17, 18], the role of farmer networks in promoting formal group leaders remains overlooked [1]. This gap in our understanding is important because, along with directly affecting farmers’ decision-making, farmer advice ties may shape adoption patterns by giving rise to leaders who can influence resource and information access. Research into how leadership and farmer networks are informed by the broader community forces can provide fruitful insights into the socio-political impacts of bottom-up extension services, as indicated by Cook et al. [1].

In this study, we use social network analysis to determine if agroforestry advice ties predict organizational leadership in an agroforestry-based farmer group in Myanmar. We ask: what are the structures of advice networks on agroforestry practices and do these networks predict organizational leadership in farmer groups?

## Theoretical framing: Farmer social networks and leadership

Since the 1970s, there has been an exponential rise in publications using social network analysis, as scholars of various fields move toward more relational and contextual explanations [19]. Social network analysis is a body of theories, methods and applications that focus on ties or relationships between social entities as nodes in a network to help explain underlying drivers of social processes and outcomes [20]. Early works of farmer networks were largely propelled by campaigns to increase the adoption of hybrid seeds, emerging alongside the diffusion of innovations theory [21–23]. More recently, social network studies in agrarian systems have placed greater emphasis on knowledge transfer, natural resource governance and decision-making [18, 24, 25]. A common thread across these studies is that not all networks are alike and ties and attributes of network actors are unalienable or, in other words, networks are entrenched within the social structures of which actors and ties exist.

However, compared to other disciplines such as business management, which has extensive literature on relational leadership formation between group members [26, 27], the agrarian field has paid scant attention to the network patterns by which leaders are produced. In South Africa, Gwiriri and Bennett found that political power and perceived connectedness were fundamental characteristics to leadership selection among five livestock cooperatives, although an empirical investigation into the network relations was missing [28]. For agroforestry management, farmers in Ghana who were highly participative in social activities were well-connected in their community networks, as indicated by their popularity to be sought after for advice [29]. Following this and findings from Gwiriri and Bennett, farmers who are central in the networks may be perceived by others as more powerful and move into leadership roles, as seen across studies in business management [26].

With leadership potentially affecting the performance of farmer groups and more of these groups undergoing democratization [11, 28, 30, 31], we addressed the aforementioned research gap by examining the structures and implications of farmer advice networks on leadership outcomes. Based on the social networks literature, we developed two hypotheses. First, we hypothesized that group members who are central in farmer advice networks on agroforestry were more likely to be endorsed as leaders. We selected ‘in-degree’ and ‘betweenness centrality’ as the network metrics of a farmer to predict leadership. As described in Table 1, in-degree centrality is the number of incoming ties for advice requests to a farmer. Betweenness centrality represents the brokerage roles of farmers based on the extent to which they lie on the shortest paths between other pairs of farmers. This concept measures farmers’ ability to coordinate and control information flow between subgroups of the network [25, 32]. Second, we hypothesized that a salient core–periphery structure exists in both the advice-seeking and advice-giving networks between members of an agroforestry-based farmer group and that leaders would be positioned in the core. Core farmers are at the center of the network by having a high density of ties among themselves while peripheral farmers are at the margins and have few ties in common [29]. This analysis illuminates network patterns shaping resource mobilization between farmers with and without leadership roles. As well, it identifies farmers who can help diffuse innovations to the larger network, necessary for the persistence of agroforestry across time.

**Table 1:** Key network measures for study hypotheses.

Measure	Description
In-degree centrality	A count of the number of ties for advice requests received by a farmer.
Out-degree centrality	A count of the number of ties for advice requests sent by a farmer.
In-degree and out-degree centralization	A measure of the concentration of ties to only a few farmers in the network by comparing the graph’s centrality measures to the idealized star network. In-degree centralization focuses on ties received and out-degree centralization focuses on ties sent.
Node betweenness	A measure of centrality that is based on the extent to which a farmer lies on the shortest paths between other pairs of farmers in the network.
Reciprocity	A measure of the number of advice requests that are reciprocated in the network.
Fragmentation	A measure of the proportion of farmers that cannot reach each other in the network.
Core-periphery structure	A categorical function that partitions actors based on the density of ties. Core actors are closely connected while periphery actors have few ties in common.

Sources: Hanneman and Riddle [33].

## The case study: Agroforestry-based farmer groups in the Ayeyarwady Delta, Myanmar

Unlike other agricultural-based economies, Myanmar has only witnessed the emergence of farmer groups since the late 2000s and early 2010s due to past legal constraints, resulting in comparatively limited analysis of local agricultural stakeholders [34]. We selected an agroforestry-based farmer group called Thone Yaung Che as the case study to examine the network effects of advice ties on organizational leadership. Thone Yaung Che means ‘three colors’ in Burmese, signifying the integration of agriculture, forestry and fishery in farming systems. This farmer group is located in the Ayeyarwady Delta and emerged from a local project that aimed to secure short-term livelihood benefits for communities affected by Cyclone Nargis in 2008. All members of Thone Yaung Che have adopted agroforestry. The members are from four surrounding villages in the Delta.

Starting in 2013, the local project staff introduced agroforestry models to Delta farmers. These models included multi-storey, intercropping, mud crab-based silvofishery, and agrisilvofishery. Thone Yaung Che formalized its organizational structure to include elections in 2018. Elections produce leaders who are part of committees in the four villages and are held at the end of each year, with the most recent one in December 2019. All members of the farmer group in the respective four villages are considered for leadership roles and do not need to register as electoral candidates. Each of the four village committees comprises four to five people who occupy positions of administrative leader, secretary, treasurer and membership advisor. While the administrative leaders influence the timeline of agroforestry activities, all committee members, regardless of their designated roles, have authority over the action plans because the farmer group employs consensus decision-making.

Farmers in leadership positions are responsible for holding quarterly meetings about the progress of agroforestry activities, allocating and monitoring loans from a revolving fund, and reporting to the larger regional farmer committee on the work plan. Thone Yaung Che has not yet established membership fees, although households interested in joining the group need to be approved by the village committee. The membership criteria include the possession of clear land rights at the community level, formal registration as a household in the government database, and a good record of repaying loans. Members have access to technical agroforestry training, free tree seedlings and crop seeds, and loans from the revolving fund to develop their agroforestry site. Group activities and resources are supported by a local non-profit organization, Green Environment and Development Association (GEDA), a legacy of the initial project.

Advice about agroforestry centers around the selection of models and species and is mostly provided through face-to-face interactions owing to the weak phone infrastructure. The main reasons for adopting agroforestry include learning new practices, increasing income, and improving food security. Farmers sought advice on optimal design and resource requirements based on their historical land use patterns and biophysical conditions. For example, before selecting a model, rice farmers with fields inundated by seasonal floods consulted with farmers who integrated fishery components into their agroforestry practices. These interpersonal communication channels helped farmers effectively use their limited resources through social learning, which informed their management decisions.

## Methodology

### Study design

**Fig 1:**
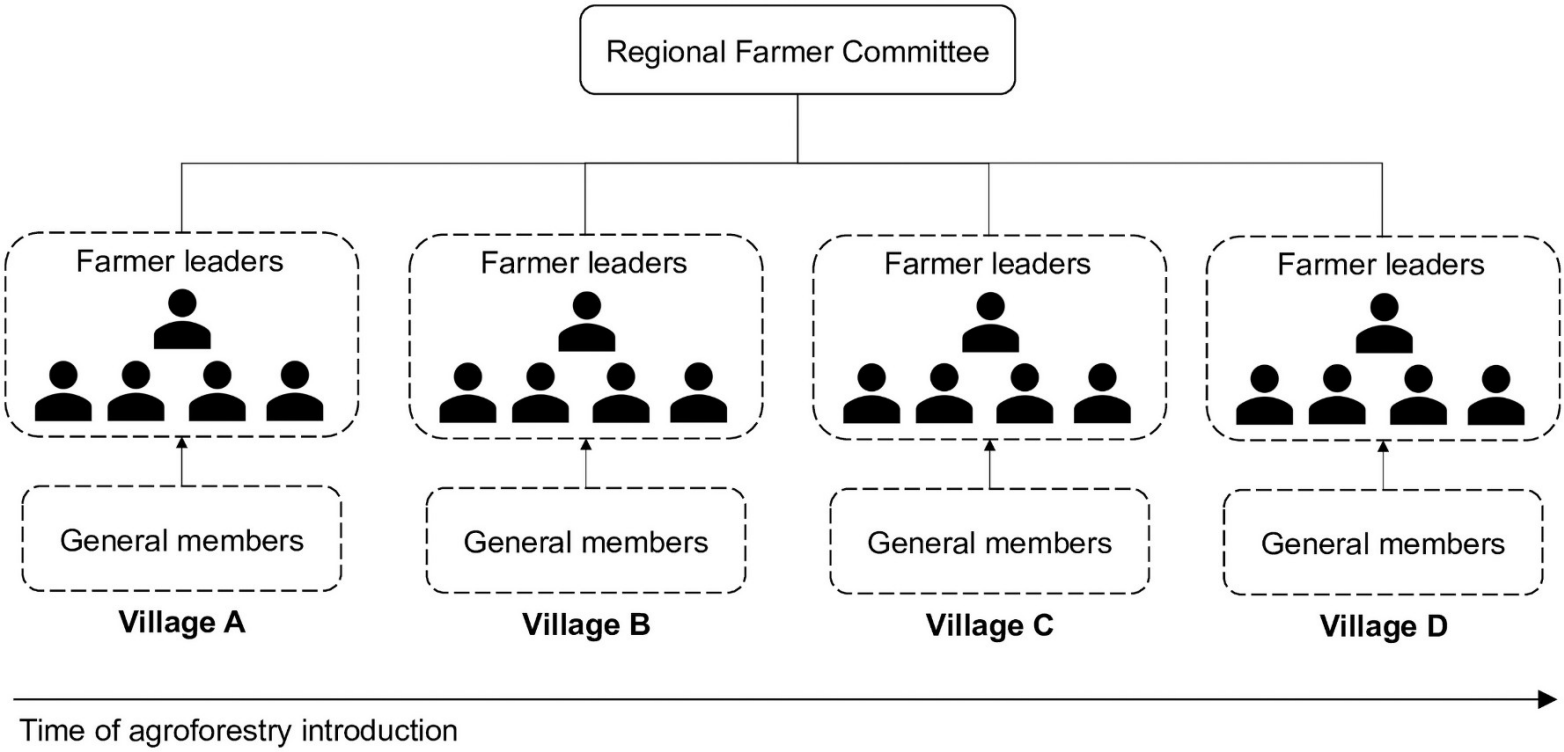
Organizational structure of Thone Yaung Che. Farmers elected into leadership positions, including the administrative leaders, are ‘farmer leaders’ represented by the person icons and farmers outside these positions are ‘general members.’ Villages are ordered by the time of agroforestry introduction, indicated by the arrow.

The four villages in our study area are labelled as village A, village B, village C and village D. Agroforestry was introduced in different years: 2013 in villages A and B, 2015 in village C, 2018 in village D. Accordingly, village A and B are sites of ‘early agroforestry introduction’, village C ‘middle’ and village D ‘late’. Based on the organizational structure of the group, we refer to farmers elected as committee members as ‘farmer leaders’ and farmers outside this group as ‘general members’ (Fig 1). As of March 2020, there were 53 members in the four villages, distributed 30.19% in village A (n = 16), 28.30% in village B (n = 15), 20.75% in village C (n = 11), and 20.75% in village D (n = 11).

### Network data collection and analysis

In this study, members of Thone Yaung Che are the nodes in the network and their relationships with other farmers to seek and give advice on agroforestry are the ties. We took a ‘whole network’ approach to capture both the presence and absence of ties between members. A threshold of 80% of the whole network was achieved through the participation of 42 of the 53 members, similar to other studies using a whole network approach [35, 36]. Data collection methods were conducted in Burmese, the primary language of all respondents.

The University of Toronto’s Human Research Ethics Unit reviewed and approved the project. The Human Ethics protocol (No. 0003857) was obtained in January 2020, in advance of data collection in March 2020. Free, prior, informed consent of study participants was obtained verbally. All participants were over the age of 18 years old.

Network data was gathered through household surveys using a roster of members. Because interested adopters typically join as a household and one household is allocated one vote in an election, we invited one adult household member from the list to participate in this study. We asked respondents if they sought advice about agroforestry from farmers in the roster (for advice-seeking ties) and if they gave advice about agroforestry to farmers in the roster (for advice-giving ties). The contact frequency of these ties was gathered by asking respondents how many times they interacted with their partner through the advice ties. We did not place time restrictions on the formation of advice ties. Attribute data—respondents’ age, education, gender, origin, labour availability, agroforestry experience, agroforestry workshop attendance, land ownership status, land size, agroforestry size, area allocation to agroforestry, and crop and tree species’ density of agroforestry area—were also gathered. We also confirmed which respondents held leadership roles (farmer leaders) or not (general members) in the farmer group.

Network data were input into adjacency matrices, containing rows of source nodes sending the tie and columns of target nodes receiving the tie [33]. We developed two matrices for advice-seeking and advice-giving ties. The first set of matrices contained binary advice relations, whether advice ties were present or absent between members, coded by 0s and 1s. The second set of matrices consisted of valued advice relations that represented contact frequency or the number of exchanges per tie. In addition to degree and betweenness centrality, we calculated network density, centralization, fragmentation, and reciprocity as described in Table 1 to compare the characteristics between advice-seeking and advice-giving ties.

Two-mode core–periphery models were applied to the advice ties to identify farmers at the core and periphery of the network. The core–periphery structure is a community structure that uses algorithmic detection in partitioning nodes that belong to the core and periphery of the network [29, 37]. Core nodes refer to farmers who are well-connected to other farmers at the network’s center and periphery nodes refer to farmers who are loosely connected at the margins. The density matrices are calculated for four areas to verify the model validity (core to core, core to periphery, periphery to periphery, and periphery to core). Models are valid if the cell value for core to core is higher than the other areas.

### Statistical analysis

To investigate the effects of advice ties on organizational leadership, we selected logistic regression as our model. It allows for predicting a binary outcome from a set of variables that may be continuous, discrete, binary or a mix of these [32]. By focusing on farmers’ centrality as an antecedent to leadership emergence, we selected farmers’ ties as the independent variables, which were expressed as i) degree centrality (in-degree and out-degree); and ii) node betweenness centrality. Accordingly, leadership status (farmer leaders or general members) was the dependent variable, represented as a binary response variable, with 1 assigned to farmers in leadership roles and 0 assigned to farmers a part of the general membership.

Two models were developed based on the inclusion of the different degree centrality measures, one calculated from binary ties and the other from valued ties. We also added dummy variables to the regression models to reduce the potential bias effects of categorical data on leadership status. These variables were: respondent’s gender (0 = female, 1 = male), age (0 = below mean age, 1 = above mean age), origin (0 = non-migrant, 1 = migrant), and education (0 = no formal education, 1 = have formal education). The lrm function in the package RMS in R was used to produce the model estimates and pseudo R^2^ values. Advice networks were analyzed in UCINET (V.6) and visualized in NetDraw. Descriptive statistics and logistic regression modelling were completed in R (V.3.6.1). Core–periphery structure identification was completed in UCINET.

## Results

### Farmer leader and general member attributes

**Fig 2:**
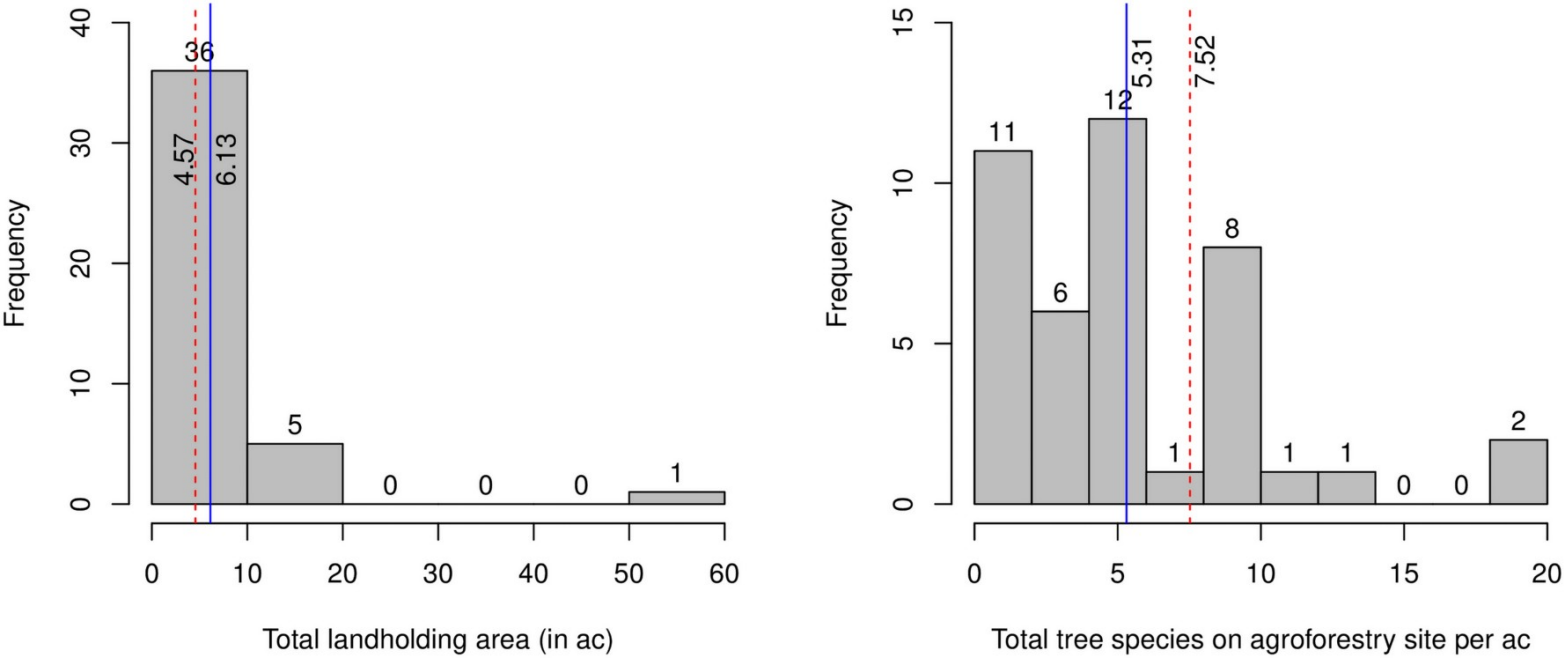
Histograms of total landholding area and tree species’ density. The dashed vertical lines in red represent the mean value of farmer leaders and the solid vertical lines in blue represent the mean value of general members.

Among the 42 respondents, 15 were farmer leaders and 27 were general members (Table 2). For the continuous socio-economic variables, farmer leaders and general members showed slight differences in total land size and tree species’ density and strong similarities in other parameters. Farmer leaders’ average total land size was 4.57 ac, compared to 6.13 ac among general members (Fig 2). While general members had larger land areas to allocate to agroforestry, farmer leaders had a higher trees species’ density (Fig 2). About 8 and 5 different tree species were planted per ac of the agroforestry site for farmer leaders and general members, respectively.

**Table 2:** Socio-economic variable definitions and descriptive statistics.

**Dependent variable**	**Definition**	**Frequency**	**%**		
Leadership status	0 (general members)	27	64.29%		
	1 (farmer leaders)	15	35.71%		
**Independent variables**		**Farmer leaders**		**General members**	
		Mean ± SE		Mean ± SE	
Socio-economic continuous					
Age	Age of respondent (years)	50.47 ± 2.71		48.85 ± 2.42	
Education	Years of formal education for respondent	7.2 ± 0.95		6.33 ± 0.68	
Labour	Number of people in household between 18–60 years old	3 ± 0.29		3.48 ± 0.26	
Experience	Years of agroforestry experience	4.07 ± 0.61		4.3 ± 0.43	
Workshop	Number of agroforestry workshops attended	2.13 ± 0.29		1.74 ± 0.26	
Farm attributes					
Agroforestry size	Total agroforestry area (ac)	0.79 ± 0.21		1.01 ± 0.19	
Land allocation	Allocation of total landholding area to agroforestry (%)	27.68 ± 6.62		33.23 ± 4.51	
Crop species’ density	Total crop species on agroforestry site per ac	9.97 ± 3.11		8.99 ± 1.92	
Socio-economic binary		Frequency	%	Frequency	%
Gender of respondent	0 (female)	1	6.67%	1	3.70%
	1 (male)	14	93.33%	26	96.30%
Origin of respondent	0 (migrant)	8	53.33%	19	70.37%
	1 (non-migrant)	7	46.67%	8	29.63%
Land ownership	0 (rent)	3	20%	1	3.70%
	1 (own)	12	80%	26	96.30%

Nearly all farmer leaders and general members have attended primary school, with the average years of schooling being 7 years for the former and 6 years for the latter. Similarly, the average age of farmer leaders was 50 years old and of general members was 49 years old. Both farmer leaders and general members have adopted agroforestry for an average of 4 years. For the binary socio-economic variables, most of the farmer leaders and general members belonged to male-headed households and owned their land.

### The structure of whole networks of agroforestry advice

**Fig 3:**
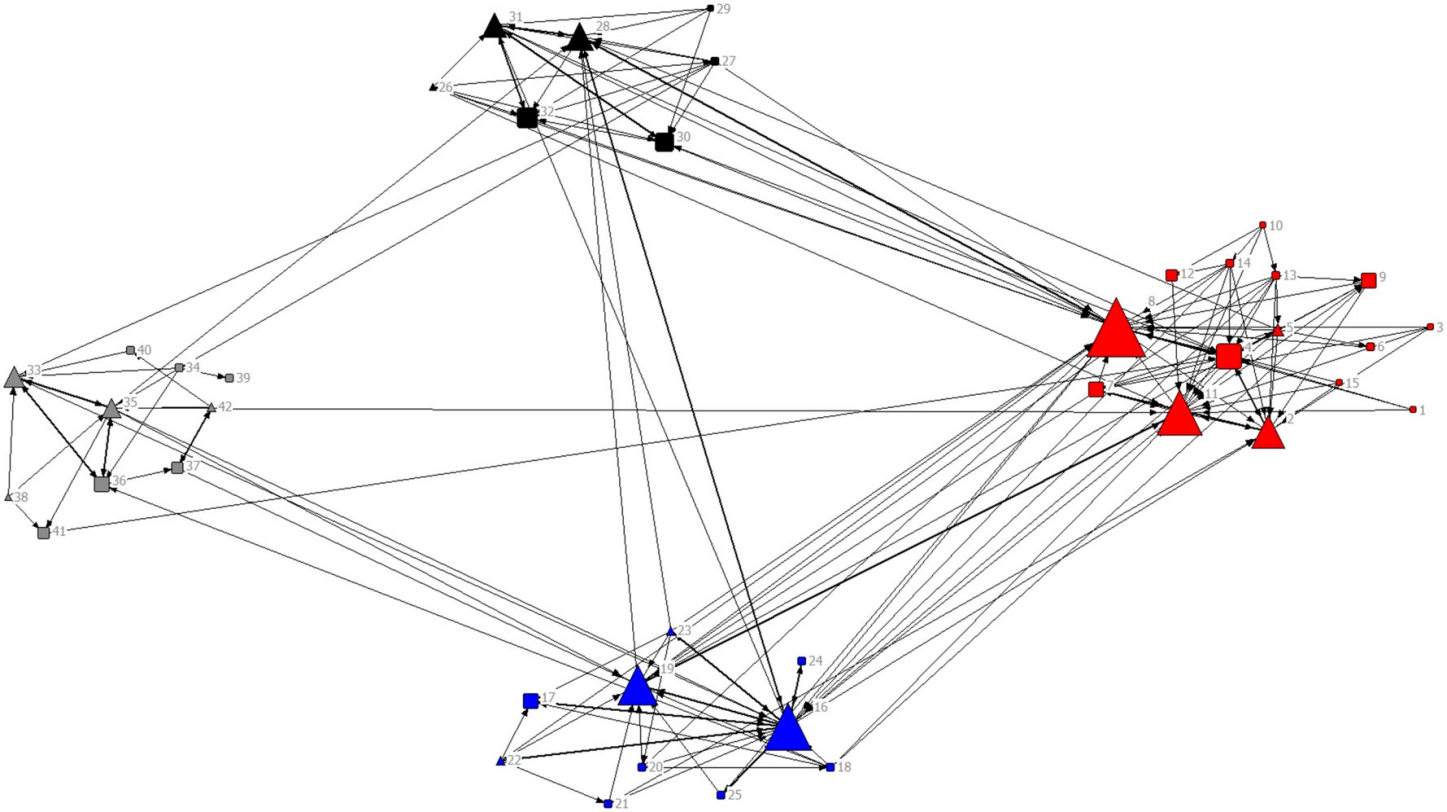
Sociogram of the advice-seeking ties. Nodes are agroforestry farmers, and ties are the relationship to seek agroforestry advice, with the arrow pointing at the person with whom they sought advice. Node colours represent farmers’ village affiliation (village A–site of early agroforestry introduction in red, village B–site of early agroforestry introduction in blue, village C–site of middle agroforestry introduction in black, and village D–site of late agroforestry introduction in grey). The node shape represents farmers’ leadership status (farmer leaders in triangles and general members in squares). The node size represents in-degree centrality, with size proportional to the value. Thicker width in edges or lines indicates reciprocated ties.

The density of the advice-seeking and advice-giving ties was the same, however, in-degree centralization for advice-seeking was 16% higher than for advice-giving (Table 3). Compared to advice-giving ties, a fewer number of farmers received advice requests. In contrast, a fewer number of farmers engaged in ties to give advice than request advice, as shown by the higher out-degree centralization score of the advice-giving network. In both the advice-seeking and advice-giving networks, most ties were not mutual, meaning that farmers reciprocated only a few advice ties. Finally, farmers with high in-degree tended to be farmer leaders (farmers 1, 2, 8, 16 and 19; Fig 3).

**Table 3:** Global measures of the advice-seeking and advice-giving networks.

Measures^a^	Advice-seeking	Advice-giving
Node	42	42
Tie	165	173
Density	0.1	0.1
Out-degree centralization	0.13	0.27
In-degree centralization	0.51	0.35
Fragmentation	0.32	0.34
Reciprocity	0.14	0.22

^a^Node betweenness score not shown in this table as measure varies for each individual farmer.

### The structural positions of farmers in advice-seeking networks on agroforestry practices

**Fig 4:**
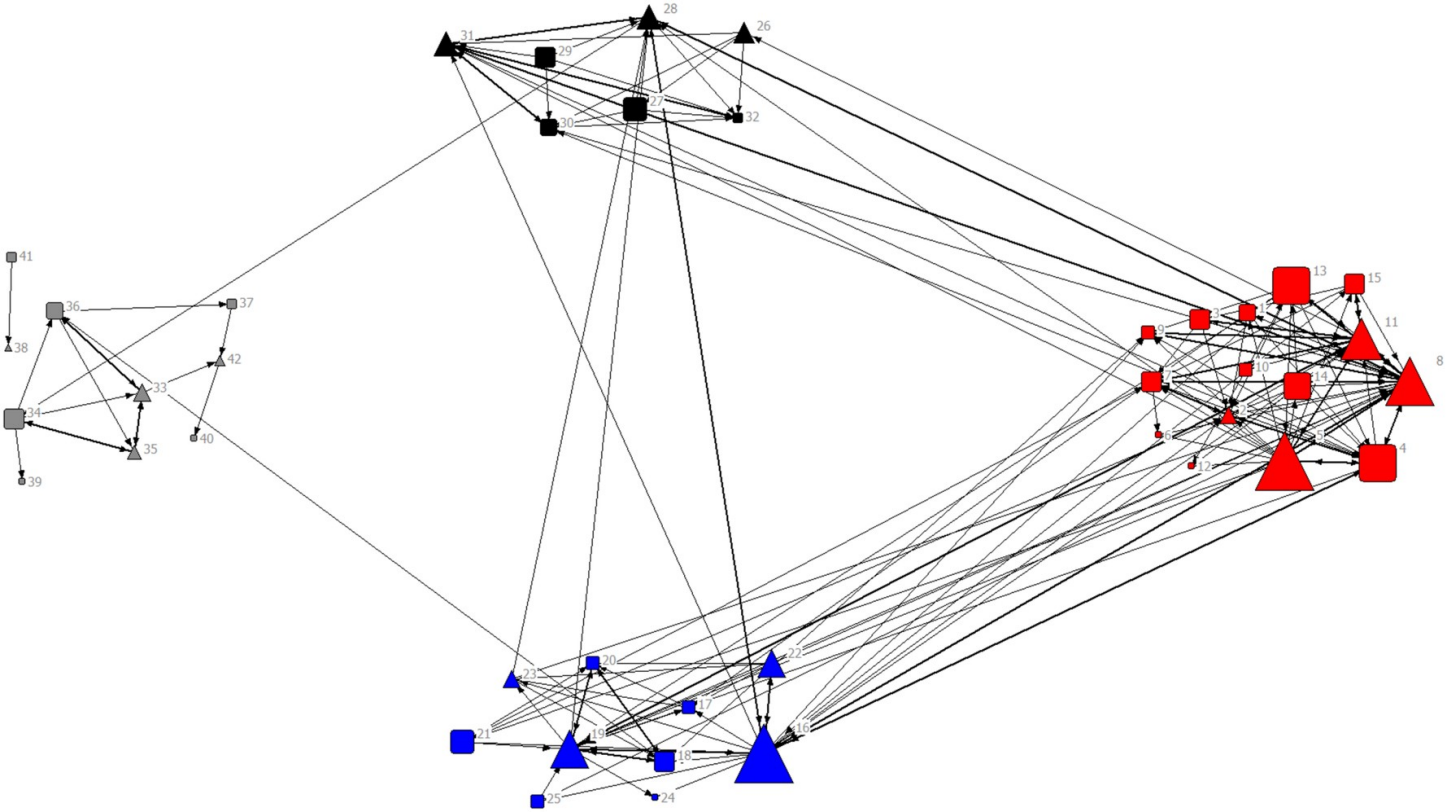
Sociogram of the advice-giving ties. Nodes are agroforestry farmers, and ties are the relationship to give agroforestry advice, with the arrow pointing at the person with whom they gave advice. Node colours represent farmers’ village affiliation (village A–site of early agroforestry introduction in red, village B–site of early agroforestry introduction in blue, village C–site of middle agroforestry introduction in black, and village D–site of late agroforestry introduction in grey). The node shape represents farmers’ leadership status (farmer leaders in triangles and general members in squares). The node size represents out-degree centrality, with size proportional to the value. Thicker width in edges or lines indicates reciprocated ties.

On average, farmer leaders received 7.5 ± 1.8 advice requests whereas general members received 2 ± 0.4 advice requests. Similarly, farmer leaders were sought out for advice more frequently (29.5 times ± 8.9) than general members (7.1 times ± 1.7). There was a much smaller range of in-degree centrality among general members than farmer leaders: the maximum advice requests being 8 for the former and 22 for the latter. However, the mean out-degree centrality of advice-seeking ties was similar between farmers with and without leadership roles (4.9 ± 0.5 for farmer leaders and 3.4 ± 0.4 for general members). Likewise, on average, farmer leaders sought advice 17.1 times ± 5.1 from their network partners and general members sought advice 14 times ± 2.8 from their network partners. The mean betweenness centrality for general members and farmer leaders was 37.6 ± 14.8 and 111.7 ± 39.3, respectively, with an outlier in the latter group (farmer 16; Fig 4).

The logistic regression models show that farmers who received more advice-seeking ties were more likely to be in leadership positions (Table 4). In the first model that used binary tie relations, all network variables were positively associated with leadership status. However, only in-degree centrality was significant (p = 0.015). The significance level of in-degree centrality in the first model was maintained when adding the dummy variables (p = 0.026). In the second model that used valued tie relations, in-degree centrality from the contact frequency of receiving advice requests was also the only significant variable (p = 0.041). However, when adding in the dummy variables, in-degree centrality was not significant (p = 0.090).

**Table 4:** Logistic regression estimates of ego farmer network measures of advice-seeking ties on the binary probability of farmers holding leadership positions.

Network variables	Model 1 (binary tie relations)	Model 1 with dummy	Model 2 (valued tie relations)	Model 2 with dummy
	Coefficient ± SE	Coefficient ± SE	Coefficient ± SE	Coefficient ± SE
Out-degree	0.314 ± 0.190	0.401 ± 0.256		
In-degree	0.287 ± 0.118*	0.305 ± 0.137*		
Out-degree frequency			0.0006 ± 0.022	-0.004 ± 0.027
In-degree frequency			0.069 ± 0.034*	0.065 ± 0.039.
Betweenness	0.0006 ± 0.005	0.005 ± 0.007	0.004 ± 0.004	0.009 ± 0.006
**Dummy variables**				
Gender		3.133 ± 2.130		2.575 ± 1.643
Age		0.656 ± 1.086		0.473 ± 0.939
Origin		1.922 ± 1.143		1.242 ± 1.016
Education		0.699 ± 1.465		0.822 ± 1.375
**Log likelihood**	15.63	23.25	11.99	17.53
**Log likelihood ratio test**	0.001	0.002	0.007	0.014
**Pseudo R2**	0.427	0.584	0.341	0.468

Significance levels denoted by ‘.’p<0.1,

‘*’p<0.05.

In addition to farmer leaders receiving more advice requests, farmers of the same village tended to have more ties with each other than with farmers of different villages (Figs 3 and 4). Between villages, advice exchanges were highest among farmers in sites of early agroforestry introduction (villages A and B), which were visually prominent in the networks. Farmers of village D shared advice only with each other. The two farmers of this village who were connected to the larger network received advice from farmers of villages B and C. We did not find a network interaction between farmers of villages A and D for sharing advice.

### Core–periphery structure of advice ties about agroforestry

**Fig 5:**
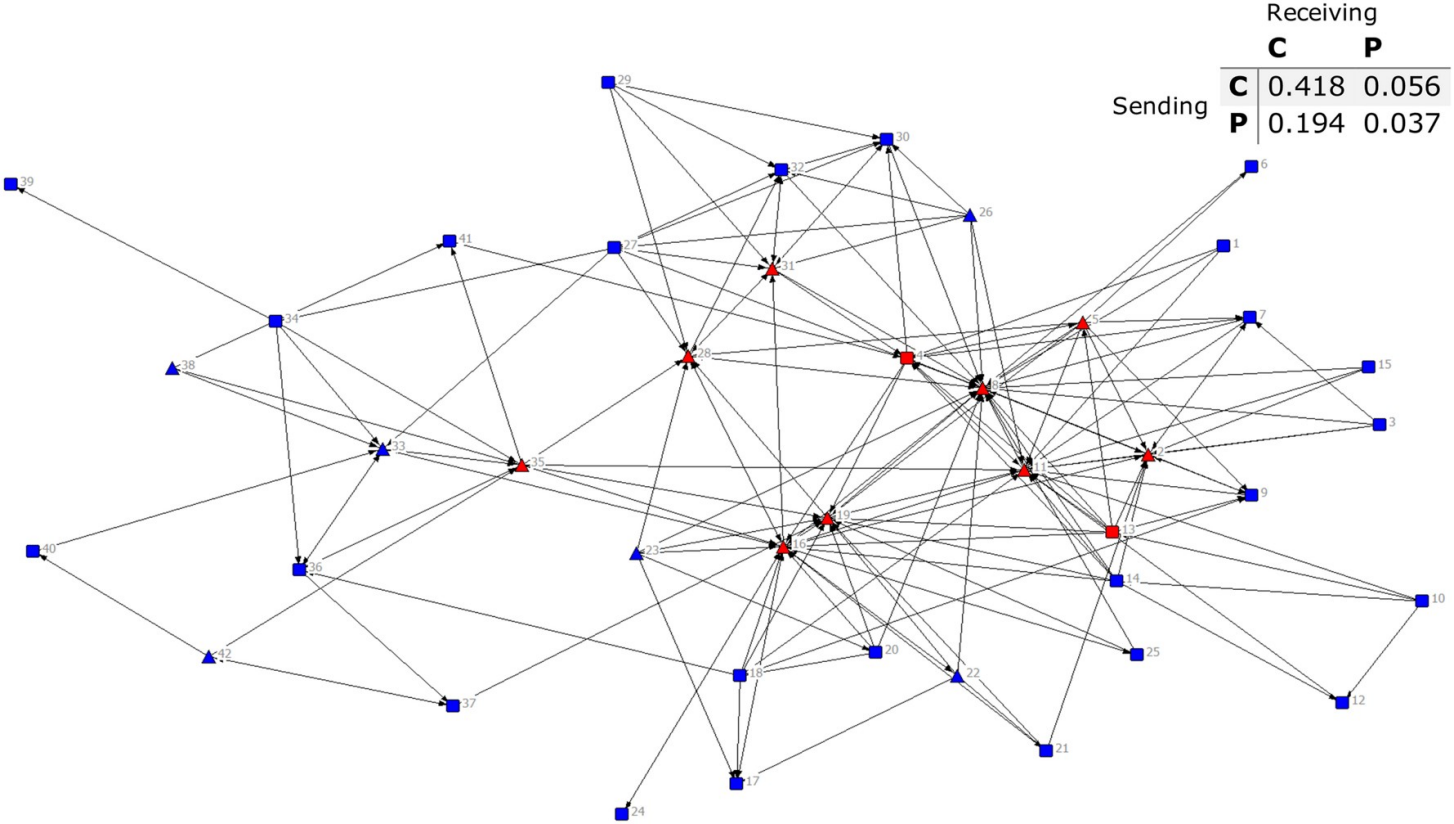
Sociogram of core-periphery membership in advice-seeking ties. The colour of nodes represents core-periphery membership (nodes in red are core farmers and nodes in blue are periphery farmers). The node shape represents farmers’ leadership status (farmer leaders in triangles and general members in squares). The density matrix of ties between core (C) and periphery (P) farmers is on the upper right-hand side of the figure.

**Fig 6:**
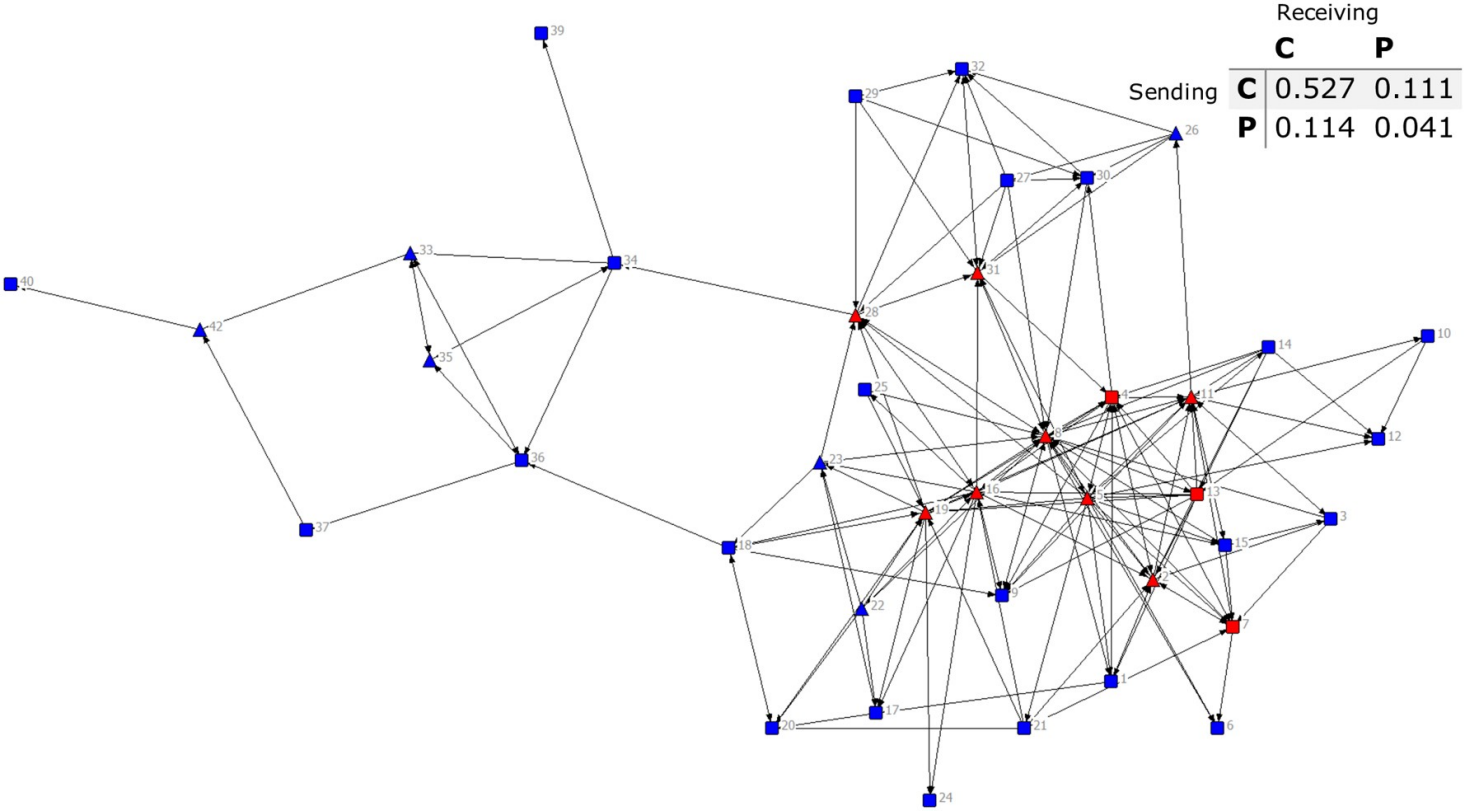
Sociogram of core-periphery membership in advice-giving ties. The colour of nodes represents core-periphery membership (nodes in red are core farmers and nodes in blue are periphery farmers). The node shape represents farmers’ leadership status (farmer leaders in triangles and general members in squares). The density matrix of ties between core (C) and periphery (P) farmers is on the upper right-hand side of the figure.

Based on core-periphery analysis, farmers in the core tended to be leaders. For instance, for both advice-seeking and advice-giving networks, 11 farmers were in the core. Two of these farmers were general members in the advice-seeking network and three of these farmers were general members in the advice-giving network (Figs 5 and 6). Among core farmers, degree centrality (in- and out-degree) ranged from 9 to 26 for advice-seeking ties and 12 to 30 for advice-giving ties. Interestingly, in the advice-seeking network, some core farmers had ties that were nearly all for seeking advice from other farmers.

Ties between core farmers were dominated by advice-giving ties rather than advice-seeking ties (C to C matrix cell values in Figs 5 and 6). Core farmers engaged in fewer ties with periphery farmers to seek advice than did periphery farmers engaged in ties with core farmers to seek advice (Figs 5 and 6). The proportion of ties sent from core to periphery farmers and vice versa was about the same for sharing advice. All four administrative leaders of the farmer group were core farmers in the advice-seeking network but only three of these leaders were core farmers in the advice-giving network. Specifically, no farmers from the site of late agroforestry adoption (village D) were at the core of the advice-giving network (Fig 6).

## Discussion

With the shift towards participatory and decentralized extension alongside the worldwide budget decline for agricultural services, more community-based farmer groups are taking on advisory roles [1, 38]. These advisory roles are central to enabling the adoption of agroforestry. Yet, little is known of local electoral processes that lead to advisory leaders and leaders in general. Here, we show that understanding advice networks can confer insights into the formation of agroforestry group leaders. Specifically, the number of advice-seeking ties, or in-degree centrality, and the frequency of these ties, positively predicted farmers’ leadership roles in an agroforestry group. While the study findings might seem evident, to date no research has examined the relationships between advice ties about agroforestry management and leadership in farmer groups. These patterns present a more nuanced assessment of the potential adoption and longevity of agroforestry practices.

This study suggests interpersonal advice ties can create pathways to leadership through the topological effects on farmers’ structural positions in the network, aligning with cases indicating leader and non-leader interactions as mutually constitutive [10, 32]. Arguably, the prestige and popularity afforded to farmers who received advice requests enabled them to be elected as leaders. In other studies, a farmer’s interconnectedness in a network, or network betweenness, was found to be significantly related to advice-seeking about agroecological management [32, 39]. However, this network metric was not a significant predictor of leadership status in our study. This might be a function of the relative newness of these farmer groups, as cliques take time to form. Furthermore, within these communities, sharing information is quite common due to the tradition of gifting [40], which may strengthen reciprocity and reduce information barriers.

Leadership emergence is often taken for granted on the assumption that those in higher socio-economic positions are more likely to acquire leadership positions [12, 13, 41]. Strong similarities and only slight differences were recorded between farmer leaders and general members among the examined socio-economic and farm variables. For instance, tree species’ density was higher for leaders rather than general members, indicating that leaders tend to adopt more intensive agroforestry practices. Other variables—such as age, education level and agroforestry experience—did not seem to affect leadership outcomes. We suspect that barriers to joining the farmer group may have reduced detectable differences between general members and leaders. Both Bryan et al. and Jerneck and Olsson describe key barriers, such as significant material and time requirements, to joining farmer groups and what this means for the adoption of agroforestry practices [7, 8]. Membership criteria may effectively bar certain farmers from even being incorporated into farmer groups, and such barriers should be considered as extension services move toward more decentralized practices.

While our primary aim was to investigate whether network metrics can predict farmers taking leadership positions, we acknowledge that the pattern of influence might be the other way around. Farmers who held elected leadership positions may have been contacted more frequently as advisors on agroforestry by virtue of their role. Although studies suggest that centrality causes influence rather than influence causing centrality [42, 43], the lack of baseline data limits the ability to rule out other potentially important antecedents to leadership emergence. Here, we focus on farmers’ advice centrality as an entry point to address this understudied topic, which we hope will encourage further analyses.

### Fostering trust through agroforestry advice-seeking

Trust may have been fostered through reciprocated actions, such as electoral support from the advice-seeker to the tie recipient, placing central farmers in more favorable positions to receive leadership endorsement. While the positive relationship between frequency of advice-seeking ties and organizational leadership was not held with dummy variables, farmer leaders were sought out for advice four times more often than general members.

The finding highlights the importance of trust between members of a farmer group for leadership emergence, which is supported by other studies that articulate the role of trusting relations in the uptake of agroforestry and agroecological technologies [3, 44, 45]. For example, Pratiwi and Suzuki found peer-to-peer advice relations were instrumental in disseminating agroforestry information in Indonesia compared to ties with friends and extension officials [44]. The rapport that farmers develop with other farmers likely improves through more frequent interactions, affecting their perception of the prototypical leader to advance the group’s goals [46].

Farmer leaders identified in this study coincide with the general description of opinion leaders. While not all opinion leaders have formal positions recognized by a group [3, 10, 32], opinion leadership parallels organizational leadership in this case because high in-degree centrality of advice-seeking ties was associated with farmers in leadership positions. Research into agroforestry advice networks has consistently shown opinion leaders as advisors within farming communities [5, 29]. Preference for opinion leaders may be high in regions where traditional cultural values are strong and highly respected [3].

### Farmer ‘brokers’ in the core of the advice networks

While farmer leaders were highly represented in the core, intra-group differences among farmer leaders also existed, with many leaders at the periphery. Unlike in-degree and out-degree centrality measures where the direction of ties is captured, core–periphery models do not account for tie direction. So, farmers who have many out-going advice ties exist in the core alongside farmers who have many incoming advice ties through the model clustering algorithm. Interestingly, core farmers who were not leaders sought and received advice requests on par with other leaders in the core membership. These farmers may act as ‘brokers’ between general members and farmer leaders in an otherwise highly concentrated environment for agroforestry information control and access.

The visible participation of general members as core farmers minimizes the power that a few farmers may have on information transfer. Brokers, or those who transfer information from one ‘group’ of farmers to another, linked leaders in the core to non-leaders or general members in the periphery, likely diffusing information and resources between these two groups. In Ghana, Isaac et al. observed that brokerage roles diversified agroforestry information through bridging experiences, which supported problem-solving and adaptation [25]. Through mediating between the core and periphery, farmer-brokers help facilitate the transfer of new, reliable information in the network and reduce emerging groupthink [25].

Farmer-brokers are not only beneficial for diffusing information across network clusters but also for sustaining adoption because general members in the core are sources of inspiration. Albizua et al. found that compared to traditional farmers who practiced sustainable farming in Spain, modern farmers were more centrally positioned in a network and controlled the information flow, leading to the spread of input-intensive farming practices [24]. Farmer-brokers in our case may offset the isolation felt by non-leader periphery farmers through enhancing and developing bonding advice ties for the persistence of agroforestry. Owing to limited government incentives for agroforestry, farmer-to-farmer outreach contributes to local capacity building.

By comparing core farmers to farmers with leadership status, we were able to assess the ability of the committees to maintain social cohesion. Although the occupation of administrative leaders in the core of the advice-seeking network suggests opportunities for enhancing cooperation across villages, this process may be constrained by interactions for giving advice. Evidently, time is needed for core–periphery structures to form. For instance, none of the farmer leaders nor general members from village D were at the core of the advice-giving network. This village was only one year out from the introduction of agroforestry, thus, had less time as compared to the other communities for the formation of a network core. Furthermore, differences in core–periphery membership between the two advice networks confirm that ties to seek advice are not equivalent to ties to give advice [47, 48].

### Policy implications for successful agroforestry adoption

Through the *ASEAN Guidelines for Agroforestry Development* [49], the Government of Myanmar is developing a national road map for agroforestry with support from World Agroforestry (ICRAF) and the Food and Agricultural Organization of the United Nations. Despite this and related activities, progress in advancing agroforestry in the country remains slow, with interventions not yet percolating down to local levels. In tandem with national reforms, attention should also be placed on farmer groups to link top–down and bottom–up approaches to agroforestry extension. The legitimacy of these groups through policy support can provide farmers with greater access to diverse knowledge sources in managing the trade-offs and synergies of agroforestry adoption. Farmer groups can also more easily form external partnerships to reduce resource constraints to adopting and scaling-up best agroforestry practices.

Strong social cohesion among farmer leaders in this study, as demonstrated by their large presence at the core of the advice networks, signals their influence and ability in mobilizing resources to achieve group goals. However, general members in the network core also indicate the valuable brokerage roles of farmers outside leadership positions in information transfer to the larger population. The presence of these members in the core may be critical to building group trust and ensuring leadership accountability, especially at the early stages of agroforestry adoption. Our insights on leadership status point to the importance of advice network analysis on assessing pathways to resource mobilization related to scaling up agroforestry.

Since farmer groups are perceived as hallmarks of democracy, local leadership composition can also be emblematic of the common voices and political concerns in a farming community [1, 15, 50]. Fostering leadership skills in members of social minorities with an emphasis on capacity building rather than the diffusion of knowledge, as suggested by Feder and Savastano, may help bridge the divide in opportunities between farmers [51]. In this process, the opportunity costs of leadership must also be acknowledged in developing interventions because many of these positions are unpaid and failure can tarnish the reputation and image of individual leaders [16]. Internal capacity building can help ensure social minorities have equal opportunities to occupy leadership positions and promote impartiality in conflict resolution.

Although our study is limited to a single case in rural Myanmar, our results could be potentially applicable to similar farmer groups. The inordinate social influence of a few network actors is commonly found in agrarian settings where the costs of service delivery and knowledge transfer are high [10, 32]. These centralized advice networks are interwoven into existing power relations and hierarchies, likely reinforcing their roles in election outcomes [10, 52]. However, organizational dynamics can be radically different from one group to another, as argued by Matous [53]. For instance, community-based farmer groups adopting similar practices may have distinct priorities, which reflect their individual members’ relationships, aspirations, resources, and environment. Internal and external ties and their degree of reciprocation highlight important contextual aspects of the group that should be understood prior to intervention [53]. The application of social network tools encourages this understanding as it moves from isolated successes of agricultural innovations and to integrated social and environmental landscapes.

## Conclusions

Local electoral processes are complex processes entwined with a myriad of socio-economic, relational, and political factors that shape one’s voting decisions. Farmers who emerge as leaders through electoral processes can greatly shape adoption behaviour by facilitating relations to share valuable information of land use management practices. Our findings contribute to understanding these processes by 1) demonstrating a positive relationship between being sought for advice ties and the propensity for members to be in elected leadership positions; 2) affirming imbalances in resource access and control through the clustering of farmer leaders at the core; and 3) highlighting the value of farmer groups to encourage adoption of new practices.

Through decentralized extension reforms, our research also shows the critical role of a farmer group in disseminating agroforestry information. This result aligns with other studies in the literature, which argue for the importance of peer advice ties in the diffusion of agricultural innovation [17, 44, 54, 55]. Serving as learning and resource platforms for agroforestry adopters, farmer groups can help fill the gap in extension support in remote areas by being a bridge between farming households and government agencies. With effective and socially inclusive leadership, farmer groups can use agroforestry to meet the vision of knowledge-intensive, sustainable agriculture.

Building on the study, we encourage investigations into other network factors, such as kinship and friendship ties, and attribute and psycho-social factors, such as personality and perception, on leadership to enrich explanations of farmers’ electoral decisions. The multi-dimensional analyses of other variables that may affect voting decisions would add greater depth in capturing leadership selection and emergence. Expanding the network boundary beyond farmer groups, to include non-adopters and extension officers, for example, can also shed light on the scalar effects of elected leaders’ prominence. Amid the decentralization of agricultural extension, these avenues for future research would be helpful to enhance the performance of farmer groups and secure broader societal goals such as sustainable land use and livelihood improvement.

## Supporting information

S1 Dataset Original network dataset. (XLS)
